# First Identification and Genomic Characterization of a Porcine Sapelovirus from Corsica, France, 2017

**DOI:** 10.1128/MRA.01049-18

**Published:** 2018-09-20

**Authors:** Géraldine Piorkowski, Lisandru Capai, Alessandra Falchi, François Casabianca, Oscar Maestrini, Pierre Gallian, Karine Barthélémy, Odile Py, Rémi Charrel, Xavier de Lamballerie

**Affiliations:** a Unité des Virus Émergents, Marseille, France; b Laboratoire de Virologie, Université de Corse-INSERM, Corte, France; c INRA, UR045 LRDE, Corte, France; d Établissement Français du Sang Alpes Méditerranée, Marseille, France; Indiana University Bloomington

## Abstract

We report the isolation and genomic characterization of a *Sapelovirus A* strain, or porcine sapelovirus (PSV), from a diarrheic Corsican piglet in France. It shares 87% nucleotide identity with a 2014 German isolate.

## ANNOUNCEMENT

Porcine sapelovirus (PSV; family *Picornaviridae*, genus *Sapelovirus*) is a 7.5- to 8.3-kb single-stranded, positive-sense polyadenylated and nonenveloped RNA virus. The genus is closely related to the genus Enterovirus and includes three species, *Avian sapelovirus*, *Sapelovirus A* (porcine sapelovirus), and *Sapelovirus B* (simian sapelovirus). It consists of a single serotype. PSV is transmitted by the fecal-oral route, and infection can be asymptomatic or associated with diarrhea, respiratory distress, encephalitis, skin lesions, and reproductive disorders (1–3). The virus has been detected worldwide in Asia (China [2], India [1], and South Korea [4, 5]), the Americas (United States [6, 7] and Brazil [8]), and Europe (Germany, United Kingdom [9], and Spain [10]).

During a campaign dedicated to identifying pigs infected by the hepatitis E virus (HEV), stool samples with positive reverse transcription-quantitative PCR (qRT-PCR) detection of the HEV genome were inoculated onto cell cultures (PLC/PRF/5 cells). Unexpectedly, a gross cytopathic effect (CPE) was observed for one specimen. It was sampled in 2017 from a 3- to 4-month-old female Nustrale (Corsican breed) piglet in a farm located in the northeast of Corsica, France. While CPE was maintained after passaging the culture supernatant, HEV was not detected, suggesting isolation of a different virus. Viral RNA was extracted from the cell culture supernatant at passage 1, using the EZ1 device and EZ1 virus minikit 2.0 (Qiagen), following the manufacturer’s instructions. Reverse transcription, nonspecific amplification, and library building were performed as previously described (11), followed by sequencing using Ion S5 technology (Thermo Fisher).

Data were treated as previously reported (12). In brief, the 95,906 reads obtained were cleaned, using CLC Genomics Workbench software v11.0.1, according to quality score (limit, 0.01) and length (reads shorter than 100 bp were removed). *De novo* assembly (requirements included the following: map reads back to contigs, mismatch cost, 2 occurrences; insertion cost, 3 occurrences; deletion cost, 3 occurrences; length fraction, 50%; similarity fraction, 80%; and minimum contig length, 350 bp) was realized, and a 7,532-nucleotide (nt)-long PSV consensus sequence, including the complete open reading frame (ORF) (7,014 nt, determined from the CLC Genomics Workbench v11.0.1 software pipeline: start codon ATG, and standard genetic code), was identified from 49,761 reads. Partial 5′ and 3′ noncoding region sequences were obtained (423/480 and 95/106 nt long, respectively, with reference to previously published PSV sequences (GenBank accession no. KJ821019, KX574284, LC326555, JX286666, and KF539414). According to current knowledge (4, 6, 13), the ORF encodes four structural proteins (VP4, VP3, VP2, and VP1) and seven nonstructural proteins (2A, 2B, 2C, 3A, 3B, 3C, and 3D). The strain was assigned the name PSV OPY-1-Corsica-2017 and made available in the European Virus Archive collection (and under GenBank accession no. MH513612).

**FIG 1:**
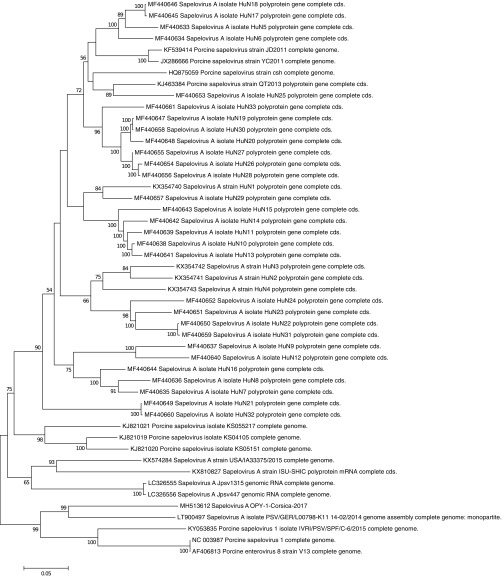
Phylogenetic tree reconstructed from PSV ORF nucleotide sequences using the maximum likelihood algorithm, 500 resampling bootstraps (GTR+G+I model, determined from the data set), and the MEGA 6.06 software.

A maximum likelihood phylogenetic reconstruction (GTR+G+I model chosen from the best model option on MEGA6 software, determined from the data set using the MEGA6 program [14]) was obtained using full ORF sequences and indicates that PSV OPY-1-Corsica-2017 is close to a 2014 German isolate (GenBank accession no. LT900497), with ca. 87% nt (6,561/7,527) and 96% amino acid (aa) (2,238/2,337) identities (Fig. 1). Phylogenetically, PSV OPY-1-Corsica-2017 clusters with Indian (KY053835) and German (LT900497, NC_003987, and AF406813) PSV isolates and is clearly separated from other strains isolated from China and the United States, among others.

In conclusion, we identified and characterized for the first time a porcine sapelovirus in France, on the Mediterranean island of Corsica. Importantly, the PSV-infected piglet from which PSV OPY-1-Corsica-2017 was isolated was born and farmed in Corsica. This is strongly suggestive of local transmission of the endemic virus.

### Data availability.

The genome sequence reported here has been deposited in the GenBank database under the accession no. MH513612.
